# Mechanistic Insights into the Non-Monotonic Flame Retardancy of CPVC/ABS Composite

**DOI:** 10.3390/polym17172415

**Published:** 2025-09-05

**Authors:** Long Zhang, Lewen Liu, Shengwen Zou, Peng Qin, Zhengzhu Zhu, Shaoyun Guo, Qining Ke

**Affiliations:** 1 Kingfa Science and Technology, Co., Ltd., Guangzhou 510663, China; zhanglong@kingfa.com.cn (L.Z.);; 2 National Key Laboratory of Advanced Polymer Materials, Sichuan University Polymer Research Institute, Sichuan University, Chengdu 610065, China; 3 School of Materials Science and Engineering, Beijing Institute of Technology, Beijing 100081, China

**Keywords:** chlorinated polyvinyl chloride/acrylonitrile–butadiene–styrene composite, flame retardancy, synergistic effect, flame-retardant mechanism

## Abstract

The chlorinated polyvinyl chloride (CPVC)/acrylonitrile–butadiene–styrene (ABS) composite represents an important class of engineering thermoplastics, offering a strong balance of flame retardancy, chemical resistance, mechanical properties, processability, and cost efficiency. Despite its widespread application, the flame-retardant mechanism in the CPVC/ABS system remains poorly understood. This work systematically investigated the non-monotonic flame-retardant behavior of CPVC/ABS composites through comprehensive characterization. The combustion performance, as determined by limiting oxygen index (LOI), UL-94 vertical burning tests, and cone calorimeter tests (CCTs), showed an unexpected pattern of flame retardancy initially improving then decreasing with reduced ABS content, which contradicted conventional expectations. The optimal composition at a CPVC/ABS ratio of 2:3 demonstrated good performance, achieving a UL-94 5VA rating and 47.3% reduction in total heat release (THR) relative to CPVC. A more stable and compact structure was observed from the morphology analysis of the residual char, and the thermogravimetric analysis further revealed a synergistic effect in carbonization behavior. The above flame-retardant mechanism could be interpreted by the combined effects of accelerated char formation during the early decomposition stage and significantly enhanced char crosslinking degree. These findings provided fundamental insights for designing high-performance flame-retardant polymer composites and facilitating their industrial implementation.

## 1. Introduction

The acrylonitrile–butadiene–styrene (ABS) terpolymer, notable for its exceptional melt-flow characteristics, notch impact strength, and surface finish quality, has emerged as a preferred material in automotive structural components, precision electronic housings, and consumer electronics [1]. However, its intrinsic flammability, manifested by a limiting oxygen index (LOI) of 18.5%, critically restricts deployment in fire-critical applications such as aircraft fields and power distribution systems. Incorporating brominated compounds [2,3], phosphorus-based systems [4,5,6], layered double hydroxides [7,8], or other features [9,10,11] into the matrix has been shown to enhance its fire inhibiting performance. However, these strategies are fundamentally constrained by ecological toxicity, substantial cost increments, and the integrated property balance of materials.

In recent years, the alloying technology of polyvinyl chloride (PVC) with ABS has garnered increasing attention due to its synergistic modification effects; the inherent chlorine content in PVC significantly enhances the flame-retardant properties of the blend, while ABS simultaneously improves the impact resistance (impact strength over 18.0 kJ/m^2^) and thermal stability (initial degradation temperature over 400 °C) of the base polymer [12,13,14,15,16]. However, the escalating industrial demands for materials with superior heat resistance and stringent UL94-V0/5VA flame-retardant certification have rendered conventional PVC/ABS systems insufficient for advanced engineering applications. Chlorinated polyvinyl chloride (CPVC), a highly chlorinated derivative of PVC with maximum chlorine content exceeding 70%, demonstrates enhanced material properties, including superior flame retardancy (LOI can reach over 60.0%) [17], elevated thermal stability (Vicat softening temperature can be over 100 °C), and improved chemical resistance (maintaining mechanical strength after 10-day acetone treatment) [18], making it a preferred choice for high-performance piping systems. Nevertheless, the extensive chlorination of CPVC leads to a dramatical increase in its melt viscosity, eventually resulting in significant challenges to its processability. The CPVC/ABS blending system presents a promising approach to address these challenges through enhancing the synergistic effects of PVC/ABS composites. Previous studies have investigated the gas-phase pyrolysis mechanism of CPVC [19,20], ABS [21,22], and CPVC/ABS composites [23], as well as their improved flame retardancy under the loading of iron-based compound [17]. Previous studies on CPVC/ABS systems have primarily focused either on analyzing the gas-phase pyrolysis behavior or on improving bulk properties such as char yield, limiting oxygen index (LOI), and smoke suppression through the incorporation of iron-based additives. However, these investigations often overlooked the critical role of formulation design and testing parameters. In particular, the influence of CPVC-to-ABS ratios and testing conditions (e.g., testing temperature) on the intrinsic flame-retardant performance has not been systematically addressed. Hence, the above investigations could provide a more comprehensive evaluation by correlating formulation composition and testing environment with key flame-retardancy benchmarks. More importantly, the flame-retardant mechanisms, especially the condensed-phase behaviors, of the CPVC/ABS still remain insufficiently understood.

In this work, a series of CPVC/ABS composites were fabricated through the melt blending method, and their combustion behavior was systematically investigated using LOI, vertical burning test (UL-94), and CCT to elucidate the flame-retardant performance. Surprisingly, the anomalous flame-retardant performance of CPVC compared to CPVC/ABS was clearly observed. More specifically, the condensed-phase flame-retardant mechanism of CPVC/ABS composites was elucidated through scanning electron microscopy (SEM), Raman spectroscopy, X-ray diffraction (XRD) spectra, and X-ray photoelectron spectroscopy (XPS) analysis, while the gas-phase flame-retardant mechanisms were illustrated by thermogravimetric analysis coupled with Fourier transform infrared spectroscopy (TGA-FTIR). The results clearly revealed the synergistic flame-retardant mechanisms between CPVC and ABS, providing a theoretical foundation for the development of high-performance flame-retardant composites.

## 2. Materials and Methods

### 2.1. Materials

CPVC (H716S, powders, chlorine content: 67%) was purchased from Kaneka Co., Ltd. (Osaka, Japan). ABS (HP181, butadiene content: 15~20%, acrylonitrile content: 20~25%, styrene content: 55~65%) was bought from Kumho Petrochemical Co., Ltd. (Seoul, Republic of Korea). Methyl tin mercaptide (liquid) was obtained from Hubei Benxing New Material Co., Ltd. (Suizhou, China). Oxidized polyethylene wax (617A, powders) was supplied by Honeywell International Inc. (Charlotte, NC, USA). Calcium carbonate (powders, particle size: 3~10 μm) was obtained from Imerys Group (Paris, France). Antioxidants (RIANOX 1010, powders) were received from Lianlong New Material Co., Ltd. (Tianjin, China). All reagents were used without further treatment.

### 2.2. Preparation of CPVC/ABS Composites

The CPVC/ABS composite pellets were prepared via melt blending using the twin-screw extrusion method. Afterwards, the UL-94, LOI, and CCT specimens were obtained by injection molding at 190 °C. The formulas of the composites are shown in Table S1. The composites were denoted as CPVC/ABS (X_1_:X_2_), where X_1_:X_2_ represented the ratio of CPVC and ABS.

### 2.3. Characterization

The LOI test was measured on a Fire Testing Technology instrument (FTT, UK) according to ISO 4582-2, and the specimen size was 120.0 × 10.0 × 4.0 mm^3^.

The UL-94 vertical burning test was performed on a CZF-5 instrument (Jiangning Analysis Instrument Company, Jiangning, China) according to ASTM D 3801. The specimen size was 127.0 × 13.0 × 1.0 mm^3^ and 150 × 150 × 1.0 mm^3^ (5VB and 5VA test).

The combustion behavior of the CPVC/ABS composites was evaluated via a cone calorimeter equipment (FTT, UK) following ISO-5660 standard at a heat flux of 25 and 50 kW/m^2^. The specimen dimensions were 100 × 100 × 3 mm^3^.

SU3400 SEM equipment (Hitachi, Japan) was employed to observe the morphology of char residues of CPVC and its composites under an accelerating voltage of 15 kV and the current of 60 μA.

The XPS spectra were acquired using Thermo Fisher 250xi equipment (Thermo Scientific, USA).

The Raman spectra were conducted via a LabRAM Aramis (HORIBA, Japan). The testing wavenumber range was from 100 to 3500 cm^−1^.

Thermogravimetric analysis (TGA) was performed on a TG 209 F3 thermogravimetric instrument (NETZSCH, Germany), with a heating rate of 20 °C·min^−1^ from 50 °C to 700 °C under a nitrogen flow of 20 mL·min^−1^.

The pyrolysis products of CPVC/ABS composites were further tested using a thermogravimetric analyzer (TG 209 F1, NETZSCH, Germany) and FTIR spectroscopy (Nicolet iS50, Thermo Scientific, USA). The results were obtained from 50 °C to 800 °C at a heating rate of 20 °C·min^−1^ (N_2_ atmosphere), and the FTIR spectra wavenumber range was from 100 cm^−1^ to 3500 cm^−1^.

The notched Izod impact test was performed using a Zwick1H IT5.5P impact tester (ZwickRoell GmbH & Co. KG, Germany) according to GB/T 1843-2008.

The tensile performance was performed with a crosshead speed of 50 mm/min on a CMT4204 testing system (Shenzhen Suns Technology Co., Ltd., Shenzhen, China) according to ASTM D412.

The Vicat softening temperature (VST) test was performed using a Compact 6 testing system (Coesfeld GmbH & Co. KG, Germany) according to ISO 306.

The melt flow rate (MFR) data was measured using a Cflow extrusion plastometer (ZwickRoell Testing Technology (Shanghai) Co., Ltd., Shanghai, China) under a load of 10 kg at 220 °C, in compliance with ISO 1133 standards.

Transmission electron microscopy (TEM) micrographs were performed using a JEM-2100F instrument (JEOL, Japan) operated at an accelerating voltage of 200 kV. The vinyl groups in ABS and CPVC/ABS were stained with osmium tetroxide to enhance contrast for clear observation.

The XRD analysis was performed using an Ultima IV X-ray powder diffractometer (Rigaku, Japan) with Cu Kα radiation. Data were collected over a 2θ range of 5–80° at a scanning rate of 5°/min.

## 3. Results and Discussion

### 3.1. Flame Retardancy of CPVC and CPVC/ABS Composites

#### 3.1.1. Limiting Oxygen Index and UL-94 Tests

**Figure 1:**
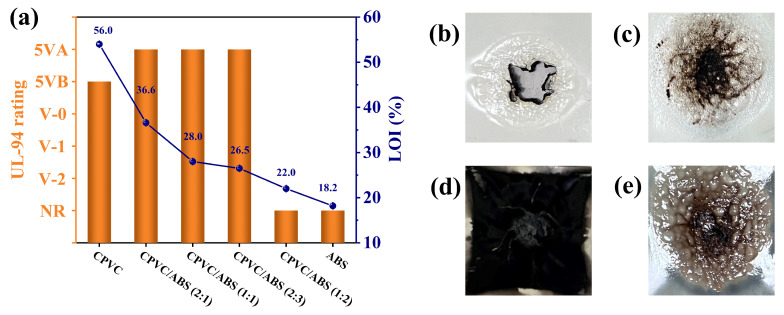
LOI and UL-94 results of the CPVC, ABS, and CPVC/ABS composites (**a**). Digital images of CPVC (**b**), CPVC/ABS (2:1) (**c**), CPVC/ABS (2:1) (**d**), and CPVC/ABS (2:3) (**e**) after UL-94 5V test.

The LOI and UL-94 tests were performed to investigate the flame retardancy of the CPVC/ABS composites. Apparently, as presented in Figure 1a, CPVC exhibited a LOI of 54.0%, indicating excellent non-combustible properties, which underscored its superior flame-retardant performance. In contrast, the pure ABS displayed flammable characteristics, and its combustion was accompanied by a melt dripping behavior as well as a low LOI (18.2%). Generally, the LOI of the CPVC/ABS composites demonstrated a progressive reduction as the CPVC content decreased. In particular, CPVC/ABS (2:1), CPVC/ABS (1:1), and CPVC/ABS (2:3) represented LOI values of 36.6%, 28.0%, and 26.5%, respectively. It is widely acknowledged that polymeric materials exhibiting LOI values exceeding 26.0% typically demonstrate self-extinguishing characteristics and achieve promising flame-retardant performance [24]. Moreover, anomalous UL-94 ratings were observed for CPVC and CPVC/ABS composites. CPVC achieved a UL-94 5VB rating, whereas CPVC/ABS (2:1), CPVC/ABS (1:1), and CPVC/ABS (2:3) attained a higher UL-94 5VA rating, demonstrating enhanced self-extinguishing properties. As illustrated in Figure 1b–d, CPVC/ABS (2:1), CPVC/ABS (1:1), and CPVC/ABS (2:3) reflected excellent flame resistance, maintaining structural integrity and continuous surface morphology. In marked contrast, the CPVC specimen experienced complete combustion penetration during the UL-94 5V test. When the CPVC/ABS ratio decreased to 1:2, the composite represented a LOI value of 22.0% and achieved no rating (NR) in the UL-94 test, indicating significantly reduced flame retardancy due to the excessive presence of flammable ABS.

#### 3.1.2. Cone Calorimeter Test

**Figure 2:**
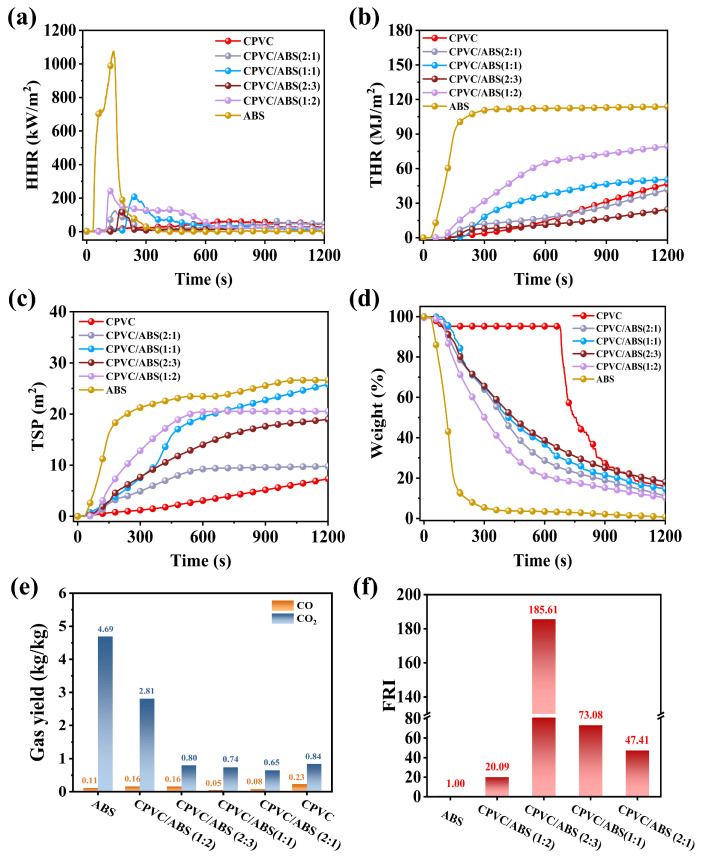
HRR (**a**), THR (**b**), TSP (**c**), and weight loss (**d**) curves of CPVC and CPVC/ABS composites. Average CO and CO_2_ yield and their ratio of the CPVC/ABS composites (**e**). FRI value of the CPVC/ABS composites (**f**).

The CCT was conducted to give an evaluation of fire control ability of the composites. Figure 2a–d present the time-dependent CCT curves, including heat release rate (HRR), total heat release (THR), total smoke production (TSP), and weight loss, while corresponding quantitative data are summarized in Table 1. Pure ABS exhibited high flammability and smoke suppression, with a peak HRR (PHRR) of 1077 kW/m^2^, a THR of 113.8 MJ/m^2^, a peak smoke production rate (PSPR) of 0.211 m^2^·s^−1^, and a TSP of 26.7 m^2^. As for the CPVC/ABS composites, the PSPR and TSP decreased as the ABS decreased, and the pure CPVC had a lowest PSPR of 0.012 m^2^·s^−1^ and TSP of 7.3 m^2^. Moreover, CPVC also exhibited a 65–178 kW/m^2^ lower PHRR than the CPVC/ABS composites. Anomalously, the THR of CPVC/ABS (2:3, 24.6 MJ/m^2^) decreased by 47.3% compared to CPVC (46.7 MJ/m^2^). However, as the CPVC content was further increased, the THR reduction for CPVC/ABS (2:1, 41.7 MJ/m^2^) was only 10.7%, implying a decline in flame retardancy. The THR (50.5 MJ/m^2^) and PHRR (210 kW/m^2^) of CPVC/ABS (1:1) were both higher than those of CPVC/ABS (2:1), despite its significantly longer time to ignition (TTI). This observation suggested that an appropriate ABS content was crucial for enhancing the fire performance of the CPVC/ABS composites, as they exhibited superior heat release suppression compared to pure CPVC. Notably, as demonstrated in Figure 2d and Table 1, the residue of ABS and CPVC were 0.8 wt.% and 15.9 wt.%, respectively. Strikingly, the residue of CPVC/ABS (2:3) was 18.1%, significantly exceeding the expected value based on individual components. This unexpected increase in residue formation indicated a clear synergistic effect in the condensed phase, vitally contributing to the improved flame-retardant properties of the composites.

The evolution of carbon dioxide (CO_2_) and carbon monoxide (CO) production could be served as critical indicators for evaluating the fire safety performance of composite materials. CO, a highly toxic gas, was generated through incomplete combustion, whereas CO_2_ represented the product of complete combustion. Previous studies illustrated that reduced emissions of both CO_2_ and CO corresponded to improved fire safety characteristics in polymeric composites [25,26]. Figure 2e displays the average CO yield (av-COY) along with the average CO_2_ yield (av-CO_2_Y) of the CPVC/ABS composites. CPVC exhibited the highest CO yield (0.23 kg/kg) among all tested materials, accompanied by a CO_2_ yield of 0.84 kg/kg. In contrast, the CPVC/ABS composites exhibited significantly lower gas emissions. Specifically, CPVC/ABS (2:1) demonstrated CO and CO_2_ yields of 0.08 kg/kg and 0.65 kg/kg, respectively. In comparison, CPVC/ABS (1:1) exhibited yields of 0.05 kg/kg (CO) and 0.74 kg/kg (CO_2_), while CPVC/ABS (2:3) produced 0.16 kg/kg of CO and 0.80 kg/kg of CO_2_. These substantial reductions in combustion products suggested enhanced fire safety performance of the CPVC/ABS composites. However, when the ABS content exceeded the optimal ratio, as observed in CPVC/ABS (1:2), the CO_2_ emissions sharply rose to 2.81 kg/kg, indicating deterioration in both flame retardancy and fire safety properties.

For a more thorough assessment of flame retardancy, the flame retardancy index (FRI) was employed as a quantitative method to evaluate the fire-resistant properties of composites [27,28,29]. The equation was as follows:

(1) $$\mathrm{FRI}=\frac{{\left[\mathrm{THR}\;\times \;\frac{\mathrm{PHRR}}{\mathrm{TTI}}\right]}_{\mathrm{Neat}\;\mathrm{Polymer}}}{{\left[\mathrm{THR}\;\times \;\frac{\mathrm{PHRR}}{\mathrm{TTI}}\right]}_{\mathrm{Composite}}}$$

The FRI-based classification could be divided into poor (FRI < 1), good (1 < FRI < 10), and excellent (FRI > 10) flame retardancy. In this study, ABS served as the neat polymer matrix (FRI = 1.00), while CPVC acted as the flame retardant. TTI referred to time to ignition, which was listed in Table 1. As illustrated in Figure 2f, the FRI showed no positive correlation with CPVC loading. Despite increasing the CPVC/ABS ratio from 2:3 to 2:1, the FRI unexpectedly decreased from 185.61 to 47.41, demonstrating that higher CPVC content compromised the flame retardancy of the composites. The CCT results reconfirmed this anomalous phenomenon, and its underlying mechanism will be systematically analyzed in the following section. A comparison of flame-retardant properties has been summarized in Table S3. Most flame-retarded ABS composites exhibit a flame retardancy index (FRI) below 10, demonstrating the significant effectiveness of CPVC as a flame retardant in ABS.

All cone calorimeter analyses discussed above were performed at 25 kW/m^2^ heat flux, representing relatively mild combustion conditions. However, under more severe conditions (50 kW/m^2^), the combustion behavior of CPVC and CPVC/ABS composites changed significantly. As evidenced by Figure S1 and Table S2, both heat and smoke release properties decreased substantially while exhibiting pronounced ABS composition dependence. Increasing ABS content led to markedly elevated PHRR, THR, PSPR, and TSP values (except for comparable THR between CPVC/ABS composites with ratios of 1:1 and 2:3). Furthermore, the TTI decreased dramatically from 277 s (neat CPVC) to 19–25 s for the composites, indicating significantly increased flammability. Naturally, the anomalous reduction in THR and increased char residue observed for CPVC/ABS (2:3) at 25 kW/m^2^ disappeared at 50 kW/m^2^. The elevated heat flux significantly accelerated the thermal degradation of the composites. Although ABS incorporation provided additional carbon sources for potential char formation, its burning features as well as the high testing temperature (~800 °C) inhibited the development of a stable char layer, consequently leading to substantially enhanced heat release under this rigorous combustion condition.

### 3.2. Analysis of the Char of CPVC and CPVC/ABS Composites

#### 3.2.1. The Morphology of Residual Char

**Figure 3:**
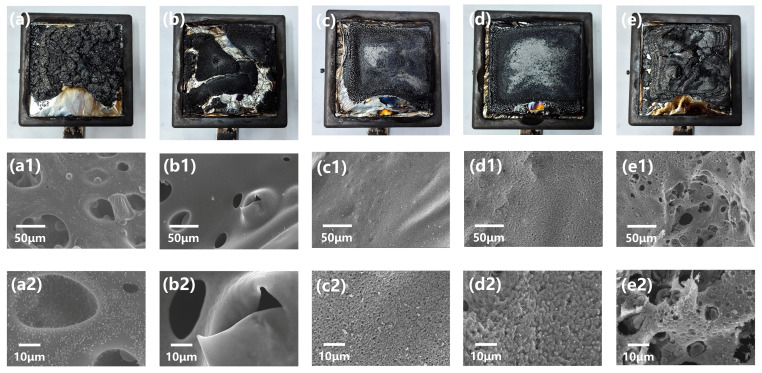
Digital photos and SEM micrographs of the char of CPVC (**a**,**a1**,**a2**), CPVC/ABS (2:1) (**b**,**b1**,**b2**), CPVC/ABS (1:1) (**c**,**c1**,**c2**), CPVC/ABS (2:3) (**d**,**d1**,**d2**), and CPVC/ABS (1:2) (**e**,**e1**,**e2**) composites after the CCTs.

SEM micrographs of the char from CCT were carried out further to acquire more details about their stability. As presented in Figure 3, CPVC/ABS (2:3) and CPVC/ABS (1:1) formed a similar dense, continuous char layer with uniform surface morphology, indicating superior char stability. In comparison, CPVC produced a continuous but highly porous char structure. CPVC/ABS (2:1) exhibited similar morphological features, though with reduced pore density and smaller pore diameters. CPVC/ABS (1:2) developed an extensively porous char network containing numerous voids, suggesting poor thermal stability. The interface of the composites before the CCTs was characterized by TEM test. To observe the dispersion of the rubber content in ABS, the vinyl groups were stained with osmium tetroxide to enhance contrast for clear observation, which has been displayed in Figure S2. When the ABS content in the CPVC/ABS composite was not high enough (CPVC/ABS = 2:1), the rubber phase of the ABS existed as isolated domains and did not form a continuous phase. In addition, a clear interface was observed between the CPVC matrix and the rubber particles of the ABS, indicating relatively poor compatibility between the two phases. In contrast, when CPVC and ABS were present in comparable proportions (CPVC/ABS = 1:1 to 2:3), the rubber content increased, resulting in a more compact dispersion of rubber domains and enhanced interfacial adhesion with the continuous phase. Due to the poor dispersion and limited compatibility of ABS, phase aggregation was promoted. Consequently, during the combustion of the CPVC/ABS blend, ABS burned in a more isolated manner, which weakened its function as a carbon source and ultimately compromised the flame retardancy of the composites.

#### 3.2.2. The Composition and Structure of Residual Char

**Figure 4:**
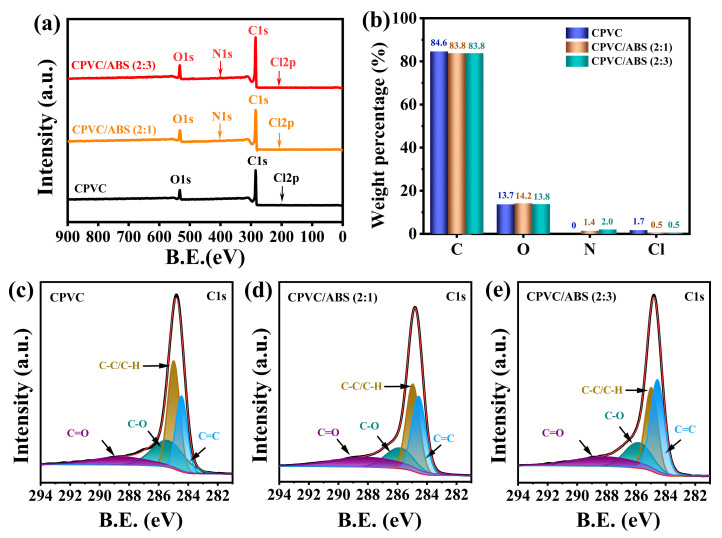
XPS spectra (**a**) and element compositions (**b**) of char residues of CPVC and CPVC/ABS composites. C1s region XPS spectra of residual char of CPVC (**c**), CPVC/ABS (2:1) (**d**), and CPVC/ABS (2:3) (**e**).

The XPS analysis of the composites char was used to further confirm their specific composition. The whole spectrum (Figure 4a) and the C1s region spectra (Figure 4c–e) of the composites as well as the weight proportion of all elements (Figure 4b), were shown in Figure 4. In addition, the O1s, Cl2p and N1s regions spectra of the composites were displayed in Figure S3. As Figure 4a,b depicted, the char spectra of CPVC and CPVC/ABS were basically alike, which displayed approximate C and O element compositions, except that little N element was observed in the char of CPVC/ABS. Notably, whether CPVC or CPVC/ABS, nearly all Cl element substance basically overflowed into the gas phase during the combustion process. The C1s spectrum demonstrated that the C element mainly existed in aliphatic and aromatic carbons (C-C/C-H: 284.7–284.8 eV, C=C: 284.0 eV) and oxidized carbons (C=O: 288.3–288.6 eV, C-O: 285.7 eV). To further analyze the thermal stability of the char residues of the composites, the Cox/Ca and C=C/C-C ratios were adopted. Here, Cox represents oxidized carbons, and Ca implies aliphatic and aromatic carbons; a lower Cox/Ca value indicates better thermal oxidation resistance of the forming char [30,31,32]. The C=C/C-C ratio, characterizing the relative abundance of unsaturated to saturated carbon bonds, serves as an indicator of char crosslinking density [33]. Typically, an elevated C=C/C-C ratio corresponds to enhanced graphitization and improved char stability. As presented in Table 2, The CPVC/ABS exhibited lower Cox/Ca values as well as higher C=C/C-C ratios compared to CPVC, suggesting enhancing thermal oxidation resistance alongside an increased crosslinking degree in the char structure. This finding warranted further investigation into the char stability differences between CPVC and CPVC/ABS composites. Notably, CPVC/ABS (2:3) demonstrated a comparable Cox/Ca ratio but a 38.7% higher C=C/C-C ratio relative to CPVC/ABS (2:1), implying the formation of a more thermally stable char residue. The O1s spectra were consistent with the C1s results, indicating that C-O and C=O groups constitute the dominant forms of oxygen in the char. In the Cl2p region, the spectrum confirmed the presence of C-Cl bonds, which could be deconvoluted into two distinct peaks corresponding to the 2p_1_/_2_ and 2p_3_/_2_ spin–orbit components. Finally, the N1s spectrum revealed that nitrogen in the char of the CPVC/ABS blend primarily exists in the form of N-H, C-N, and C=N functional groups.

**Figure 5:**
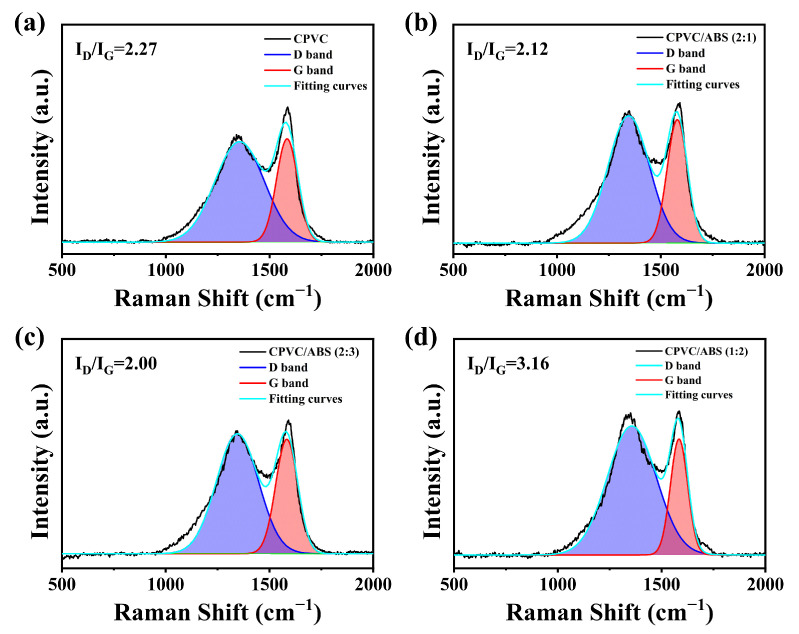
Raman spectra of char residues of CPVC (**a**), CPVC/ABS (2:1) (**b**), CPVC/ABS (2:3) (**c**), and CPVC/ABS (1:2) (**d**).

Raman spectra were employed to further elucidate the char stability of the composites. Figure 5 shows the experimental and fitting curves of the char of CPVC, CPVC/ABS (2:1), and CPVC/ABS (2:3) following the CCT. As the spectra indicate, the D band (around 1351 cm^−1^) and G band (around 1588 cm^−1^) were clearly observed, which corresponded to the vibrations of disordered carbon structure and graphitized composition, respectively [34,35]. A lower peak area ratio of D band to G band (I_D_/I_G_, defined as R) reveals a higher degree of graphitization of the char. The R value decreased from 2.27 for neat CPVC char to 2.12 and 2.00 for CPVC/ABS composites with ratios of 2:1 and 2:3, respectively. However, a notable increase to 3.16 was observed for the composite with a ratio of 1:2. The results revealed that CPVC/ABS (2:3) resulted in good char stability, followed by CPVC/ABS (2:1), with CPVC/ABS (1:2) showing the lowest stability.

The XRD spectra were employed to further investigate the structural characteristics of the char residues from the composites. As shown in Figure S4, the char derived from CPVC and CPVC/ABS (1:2) exhibited a predominantly amorphous structure, with nearly no crystalline phases detected. In contrast, two distinct diffraction peaks at 2θ = 36.9° and 43.2° were observed in the chars of CPVC/ABS (2:1) and CPVC/ABS (2:3), indicating the presence of graphitized carbon. Moreover, the crystallinity of the char from CPVC/ABS (2:3) was calculated to be 15.5%, which is higher than that of CPVC/ABS (2:1). These findings are consistent with the results obtained from CCT and Raman spectroscopy.

### 3.3. Degradation Behavior of CPVC and CPVC/ABS Composites

#### 3.3.1. Thermal Stability of CPVC and CPVC/ABS Composites

**Figure 6:**
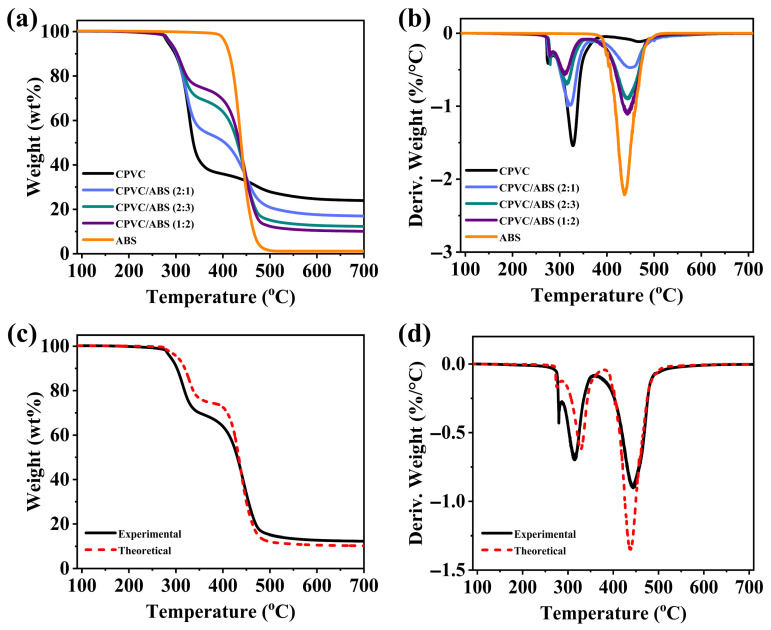
TGA (**a**) and DTG (**b**) thermograms of the CPVC, ABS, and CPVC/ABS composites under N_2_ atmosphere. Experimental and theoretical TGA (**c**) and DTG (**d**) curves of CPVC/ABS (2:3).

TGA was applied to demonstrate the synergistic effect between CPVC and ABS concerning the thermal stability of the CPVC/ABS composites. Figure 6a,b and Table 3 present the TGA/DTG curves and corresponding data for CPVC and CPVC/ABS composites in N_2_ atmosphere. The CPVC initially degraded at 283 °C (T_d,5%_), exhibiting two distinct maximum degradation temperatures (T_max1_ and T_max2_) at 328 °C and 470 °C, respectively, leaving a residual char yield of 23.9%. In contrast, pure ABS demonstrated a higher initial decomposition temperature (405 °C) and rapidly attained its peak mass loss rate at 437 °C, yielding only 1.1% residue. The CPVC/ABS composites displayed a combined degradation behavior, retaining CPVC-like T_d,5%_ and T_max1_ values while shifting T_max2_ toward the ABS-dominated regime. Additionally, increasing the ABS content led to a progressive rise in first maximum mass loss rate (MLR_max1_) but a concurrent decline in second maximum mass loss rate (MLR_max2_). Notably, CPVC/ABS composites demonstrated significantly higher experimental residue values compared to theoretical predictions. For instance, at 60% ABS loading, the experimental residue reached 12.3%, exceeding the theoretical value of 10.3% by 20.6%, indicating a pronounced synergistic effect in char formation. To elucidate this synergy, Figure 6c,d present comparative experimental and simulated TGA/DTG curves for CPVC/ABS (2:3), where the simulation was derived from weighted summation of individual component curves. Apparently, the experimental data revealed accelerated decomposition in the first stage (~250–320 °C) but delayed decomposition in the second stage (~350–450 °C), accompanied by a 33.8% reduction in maximum mass loss rate (MLR_max_). This behavior suggested that enhanced early-stage decomposition facilitated more extensive char formation at lower temperatures, which subsequently inhibited further degradation at elevated temperatures.

However, previous studies have reported an antagonistic char-forming effect between ABS and other polymer matrices, including PVC/ABS and PC/ABS composites [12,36]. Moreover, little residual char (no more than 2.0 wt.%) was found after the thermal degradation (at 700 °C) of the PVC/ABS composites. Distinctly, this phenomenon where the flammable ABS component significantly compromised the char-forming capability of PVC (or PC) was not observed in the CPVC/ABS system.

#### 3.3.2. Analysis of the Gas Phase Pyrolysis Products

**Figure 7:**
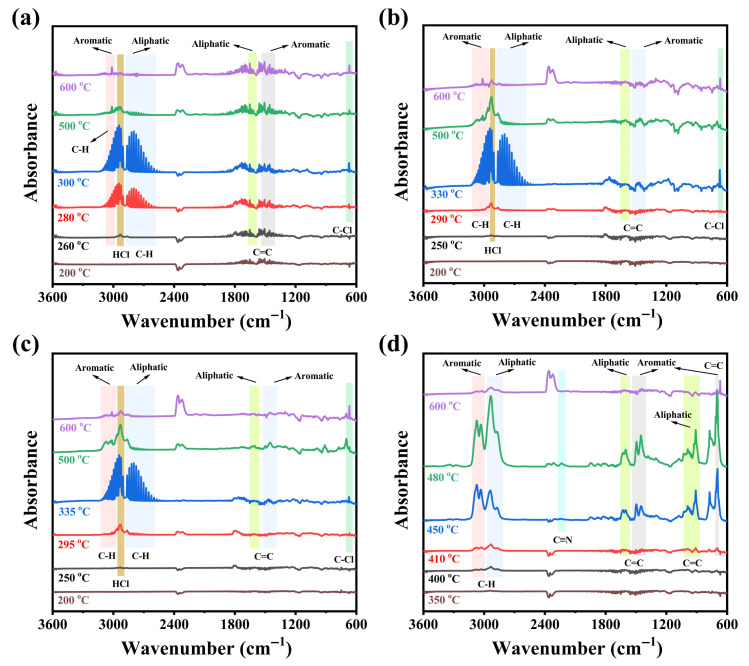
TG-FTIR spectra of CPVC (**a**), CPVC/ABS (2:1) (**b**), CPVC/ABS (2:3) (**c**), and ABS (**d**).

TG-FTIR spectroscopy was employed to identify the gaseous products evolved during thermal degradation, as detailed in Figure 7. As for CPVC (Figure 7a), most gaseous products initially appeared at 260 °C, primarily consisting of hydrogen chloride (HCl, at 2970 cm^−1^), along with characteristic absorption peaks for aromatic C-H (1615–1800 cm^−1^), aliphatic C-H (at 1615–1800 cm^−1^), aromatic C=C (at 1615–1800 cm^−1^), and aliphatic C=C (at 1615–1800 cm^−1^). Subsequently, the C-Cl stretching vibration (668 cm^−1^) became detectable at 280 °C. The maximum evolution of HCl and hydrocarbon occurred at 300 °C, reflecting the dramatic degradation of CPVC. For pure ABS (Figure 7d), the initial decomposition products (aromatic and aliphatic C-H/C=C) appeared at 400–410 °C. A weak C≡N peak, indicative of hydrogen cyanide release, was detected at 450 °C. Afterwards, the maximum release of the C=C/C-H groups occurred at 480 °C, completing the major decomposition gas release.

The CPVC/ABS composites (Figure 7b,c) exhibited nearly identical gas evolution information to pure CPVC, yet with notable differences in thermal degradation behavior. Specifically, the initial release of volatile compounds occurred approximately 10 °C earlier, while the peak HCl emission was delayed by 30–35 °C compared to CPVC. This suggested that the flammable ABS component promoted the early-stage decomposition but subsequently suppressed further degradation of CPVC. Moreover, the C=C/C-H groups intensities in the CPVC/ABS composites reached their maximum at 330–335 °C, a temperature higher than that of pure CPVC (300 °C) but substantially lower than that of neat ABS (480 °C). These observations clearly demonstrated that the compounding system retarded the thermal degradation of CPVC while simultaneously accelerating the decomposition of ABS.

### 3.4. Flame-Retardant Mechanism of CPVC/ABS Composites

**Figure 8:**
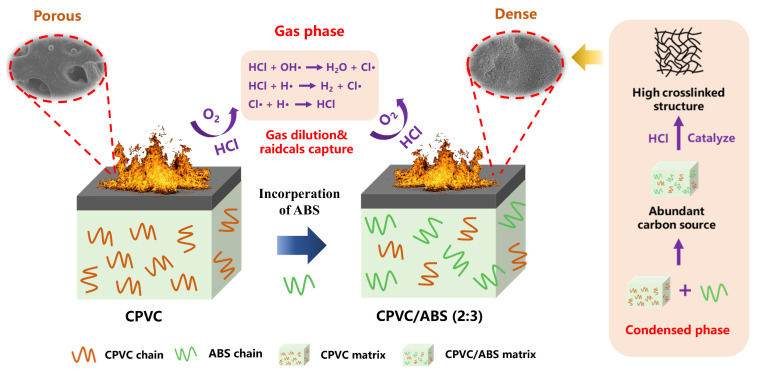
Flame-retardant mechanism of the CPVC/ABS composites.

As the above results were comprehensively described, we proposed a synergistic flame-retardant mechanism of the CPVC/ABS composites, which is presented in Figure 8. When CPVC was ignited alone, heavy HCl was produced immediately, which could capture the hydrogen and hydroxyl radicals in the gas phase, thus prohibiting flame combustion contributes to a ‘flame poisoning effect’ [37]. However, the XPS elemental analysis (cf. Figure 4b) revealed minimal chlorine participation in the final char formation. While the substantial HCl release could theoretically promote crosslinked char development, it simultaneously accelerated the thermal decomposition of CPVC [19,38]. This dual effect, combined with the inherently low carbon content (˂33 wt.%) of CPVC, resulted in an incomplete and discontinuous protective char layer that ultimately compromised the flame inhibition capability of CPVC.

For the CPVC/ABS (2:3) composite, the ABS component provided abundant hydrocarbon and phenyl groups, serving as potential carbon sources for char formation. Crucially, the flammable nature of ABS, combined with the Lewis acid catalytic effect of HCl, promoted early-stage char formation and crosslinking reactions (cf. Figure 6d and 7). This process yielded a highly crosslinked (cf. Table 2) and thermally stable (cf. Figure 4b) surface barrier, which effectively inhibited further composite degradation. Consequently, the system exhibited a pronounced synergistic effect in the condensed phase, as evidenced by the data in Table 3. Lastly, it is worth noting that insufficient ABS content (i.e., CPVC/ABS = 2:1) resulted in limited enhancing flame retardancy. Conversely, excessive ABS content compromised the flame-retardant system due to its inherent flammability, disrupting the synergistic effects observed at optimal compositions.

### 3.5. Comprehensive Performance of CPVC/ABS Composites

**Figure 9:**
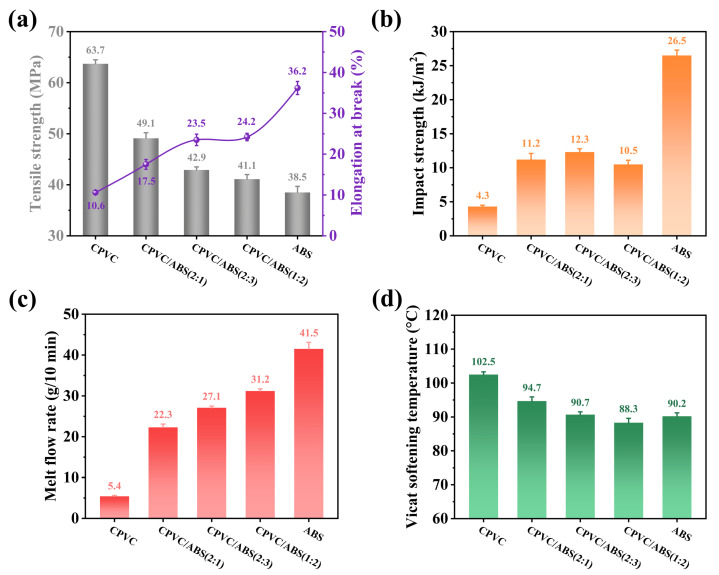
Tensile performance (**a**), impact strength (**b**), melt flow rate (**c**), and Vicat softening temperature (**d**) of the CPVC, ABS, and CPVC/ABS composites.

The results of tensile strength, elongation at break, impact strength, MFR value, and Vicat softening temperature of CPVC, ABS, and their composites are shown in Figure 9. The CPVC represented maximum tensile strength (63.7 MPa) and high VST (102.5 °C) but brittle failure (10.6% of elongation at break and 4.3 kJ/m^2^ of impact strength) and a low MFR value (5.4 g/10 min). In contrast, ABS provided superior ductility (36.2% of elongation at break and 26.5 kJ/m^2^ of impact strength) and high MFR value (41.5 g/10 min) but lower tensile strength (38.5 MPa) and Vicat softening temperature (90.2 °C). The CPVC/ABS composites exhibited intermediate properties that balanced these characteristics. The systematic investigation presented in Figure 9 revealed that increasing ABS content enhanced material toughness and processability while gradually compromising tensile strength and heat resistance. Interestingly, as depicted in Figure 9b, the impact strength of CPVC/ABS (1:2) was slightly inferior to that of CPVC/ABS (2:3), potentially attributable to the decreasing compatibility between CPVC and ABS at higher ABS loadings. Optimal comprehensive performance seemed to be achieved at ABS contents between 33 and 60 wt.%, where the composites maintained satisfactory flame retardancy while exhibiting balanced mechanical, processing, and heat resistance properties. Furthermore, Figure 9d illustrated that CPVC/ABS (1:2) displayed marginally lower VST than neat ABS, which may be ascribed to the plasticizing effect of the liquid methyl tin mercaptide heat stabilizer (4 phr loading) employed in the composite formulation.

## 4. Conclusions

This study investigated the flame retardancy of CPVC/ABS composites and elucidated the possible underlying working mechanisms. Interestingly, the CPVC/ABS composites demonstrated complex and non-linear flame-retardant behavior arising from synergistic interactions between the components. At a CPVC/ABS ratio of 2:1, the composites exhibited obvious improvements in fire performance compared to CPVC, achieving a superior UL-94 rating (5VB–5VA) along with reduced THR, CO_2_, and CO emissions. As the CPVC/ABS ratio increased to 1:1, 5VA rating was maintained and a dense char layer could be detected. More remarkably, when the CPVC/ABS ratio was adjusted to 2:3, these flame-retardant properties were not only maintained but further enhanced, with a particularly notable reduction in THR (decreased by 47.3%). These observed enhancements in flame retardancy were primarily attributed to the promotion of early-stage char formation and the retardation of subsequent degradation processes, which has been proved due to the TGA and TGA-FTIR results. Specifically, the CPVC/ABS composites could exhibit a decrease of approximately 20–30 °C in the maximum decomposition temperature, a 33.8% reduction in the MLR_max_, and a 20.6% higher experimental char residue compared to the theoretical value. The synergistic effect of these mechanisms ultimately led to the formation of a more stable and crosslinked char residue, which significantly improved the overall fire performance. Furthermore, comprehensive evaluation of the mechanical properties, processability, and heat resistance performance revealed that ABS contents ranging from 33 to 60 wt.% yielded optimally balanced performance in the CPVC/ABS composites.

The findings of this study provided critical mechanistic insights into the synergistic interactions affecting flame retardancy in CPVC/ABS composites. This study also presented a promising strategy for developing advanced flame-retardant polymer composites, especially for the thin-walled UL-94 5VA materials.

## Figures and Tables

**Table 1:** Cone calorimeter data of CPVC, ABS, and CPVC/ABS composites.

Sample	ABS	CPVC/ABS (1:2)	CPVC/ABS (2:3)	CPVC/ABS (1:1)	CPVC/ABS (2:1)	CPVC
TTI (s)	31	98	151	196	63	631
PHRR (kW m^−2^)	1077	243	131	210	126	65
THR (MJ m^−2^)	113.8	79.4	24.6	50.5	41.7	46.7
PSPR (m^2^·s^−1^)	0.211	0.130	0.084	0.101	0.055	0.012
TSP (m^2^)	26.7	20.5	18.9	25.8	9.8	7.3
Residue (wt.%)	0.8	11.2	18.1	14.7	10.3	15.9

**Table 2:** Peak intensity ratios of char residues of CPVC and CPVC/ABS composites from C1s peak fitted signals.

Sample	Cox/Ca	C=C/C-C(C-H)
CPVC	0.62	0.66
CPVC/ABS (2:1)	0.55	0.93
CPVC/ABS (2:3)	0.53	1.29

**Table 3:** TGA and DTG data of the CPVC, ABS, and CPVC/ABS composites under N_2_ atmosphere.

Sample	T_d,5%_ (°C)	T_max1_ (°C)	MLR_max1_ (%/°C)	T_max2_ (°C)	MLR_max2_ (%/°C)	Residue (wt.%)	
						Theoretical	Experimental
CPVC	283	328	1.54	470	0.12	/	23.9
CPVC/ABS (2:1)	287	317	1.00	450	0.47	16.4	17.0
CPVC/ABS (2:3)	289	314	0.69	444	0.90	10.2	12.3
CPVC/ABS (1:2)	288	311	0.57	442	1.10	8.6	10.1
ABS	405	437	2.21	/	/	/	1.1

## Data Availability

The original contributions presented in this study are included in the article/Supplementary Material. Further inquiries can be directed to the corresponding author(s).

## Supplementary Materials

The following supporting information can be downloaded at: https://www.mdpi.com/article/10.3390/polym17172415/s1, Figure S1: HRR (a), THR (b), TSP (c), and weight loss (d) curves of CPVC and CPVC/ABS composites under a heating flux of 50 kW. Figure S2: TEM micrographs of the surface of CPVC/ABS (2:1) (a, a1, a2), CPVC/ABS (1:1) (b, b1, b2), CPVC/ABS (2:3) (c, c1, c2), and ABS (1:2) (d, d1, d2) composites before the CCTs. Figure S3: O1s, Cl2p, and N1s regions XPS spectra of residual char of CPVC (a, d), CPVC/ABS (2:1) (b, e, g), and CPVC/ABS (2:3) (c, f, h). Figure S4: XRD spectra of the char of CPVC, CPVC/ABS (2:1), CPVC/ABS (2:3), and ABS (1:2) composites.; Table S1: Formulas of the CPVC, ABS, and CPVC/ABS composites. Table S2: Cone calorimeter data of CPVC and CPVC/ABS composites under a heating flux of 50 kW. Table S3: Flame-retardant comparison for CPVC/ABS composites and flame-retarded ABS according to the reported literatures. References [39,40] are cited in the supplementary materials.
