# Supplementary material for: Mechanistic Insights into the Non-Monotonic Flame Retardancy of CPVC/ABS Composite
Source: Polymers (Basel). 2025 Sep 5;17(17):2415. doi: 10.3390/polym17172415 (PMC12431173; doi:10.3390/polym17172415)
Supplement: Supplementary file 1 [file polymers-17-02415-s001.zip › polymers-3833225-supplementary.pdf]

Supplementary Materials for

Mechanistic Insights into the Non-Monotonic Flame Retardancy of CPVC/ABS Composite

Long Zhang[1,2], Lewen Liu[1], Shengwen Zou[1], Peng Qin[1], Shaoyun Guo[2,*], Qining Ke[1,3,*]

[1] *Kingfa Science and Technology, Co., Ltd., Guangzhou, China.*

[2] *Sichuan University Polymer Research Institute, National Key Laboratory of Advanced Polymer Materials, Sichuan University, Chengdu, China*

[3] *School of Materials Science and Engineering, Beijing Institute of Technology, Beijing, China.*

[*] Corresponding author: Qining Ke (E-mail: keqining@kingfa.com.cn) and Shaoyun Guo (E-mail: nic7702@scu.edu.cn)

Table S1. Formulas of the CPVC, ABS, and CPVC/ABS composites.

Sample	CPVC	ABS	CPVC/ABS (2:1)	CPVC/ABS (1:1)	CPVC/ABS (2:3)	CPVC/ABS (1:2)
H716S	100	0	66.7	50	40	33.3
HP181	0	100	33.3	50	60	66.7
Methyl tin mercaptide	4	0	4	4	4	4
Oxidized polyethylene wax	3	0	3	3	3	3
Calcium Carbonate	3	0	3	3	3	3
Antioxidants	0.5	0	0.5	0.5	0.5	0.5

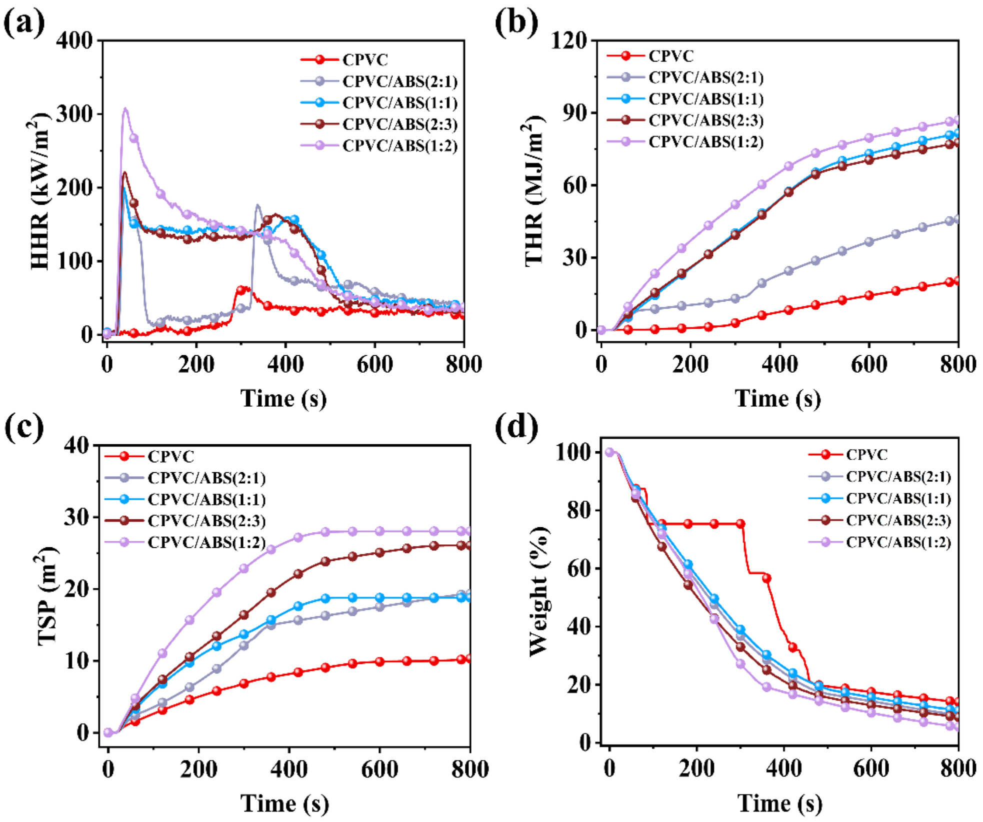

Figure S1. HRR (a), THR (b), TSP (c), and weight loss (d) curves of CPVC and CPVC/ABS composites under a heating flux of 50 kW.

Table S2. Cone calorimeter data of CPVC and CPVC/ABS composites under a heating flux of 50 kW.

Sample	CPVC	CPVC/ABS (2:1)	CPVC/ABS (1:1)	CPVC/ABS (2:3)	CPVC/ABS (1:2)
TTI (s)	277	25	24	23	19
PHRR (kW m^{-2})	65	191	200	221	308
THR (MJ m^{-2})	20.4	46.0	81.5	77.6	87.0
PSPR ($m^2 \cdot s^{-1}$)	0.057	0.110	0.121	0.148	0.150
TSP (m^2)	10.3	19.4	18.8	26.1	28.0
Residue (wt.%)	14.0	9.5	11.0	8.8	5.5

Table S3. Flame-retardant comparison for CPVC/ABS composites and flame-retarded ABS according to the reported literatures.

Sample	UL94 rating	PHRR (kW m^{-2})	THR (MJ m^{-2})	FRI	Ref
ABS+Br+CaP+8Talc	V0(1.6mm)	141	51.0	14.50	[1]
FRABS	V0(3.2mm)	383	84.0	3.89	[2]
ABS/1% d-LM@MX	/	714	95.8	2.93	[3]
24ADP@SiR/ABS	V0(3.2mm)	458	54.4	4.11	[4]
ABS-FR2-PER-MH3	V0(1.5mm)	314	96.0	11.89	[5]
CPVC/ABS (2:3)	5VA(1.0mm)	131	24.6	185.61	This work

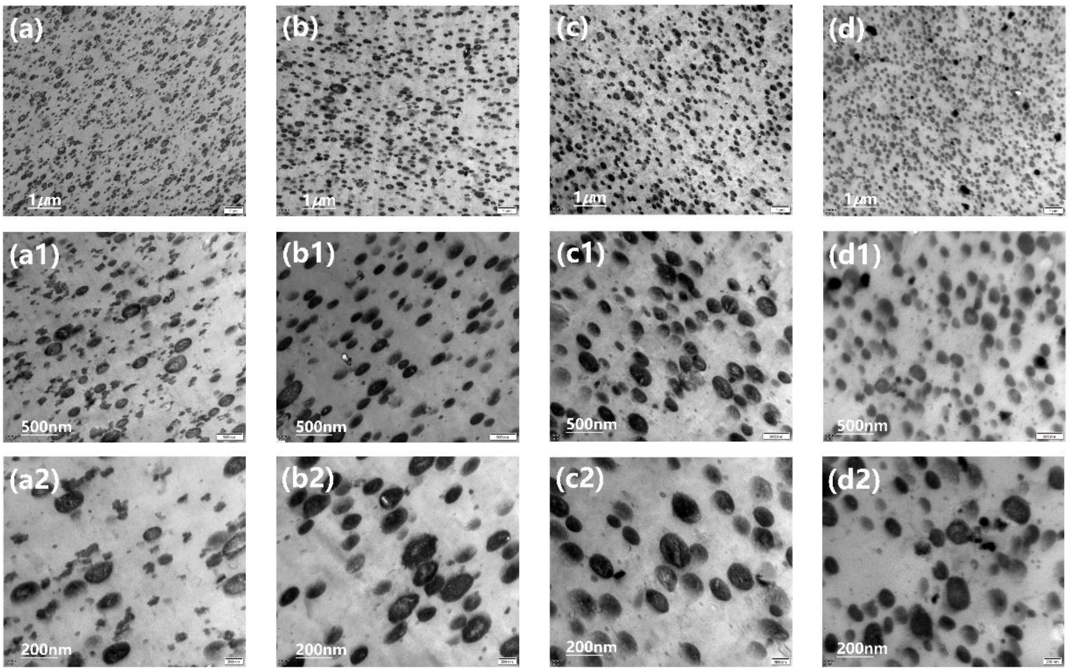

Figure S2. TEM micrographs of the surface of CPVC/ABS (2:1) (a, a1, a2), CPVC/ABS (1:1) (b, b1, b2), CPVC/ABS (2:3) (c, c1, c2), and ABS (1:2) (d, d1, d2) composites before the CCTs.

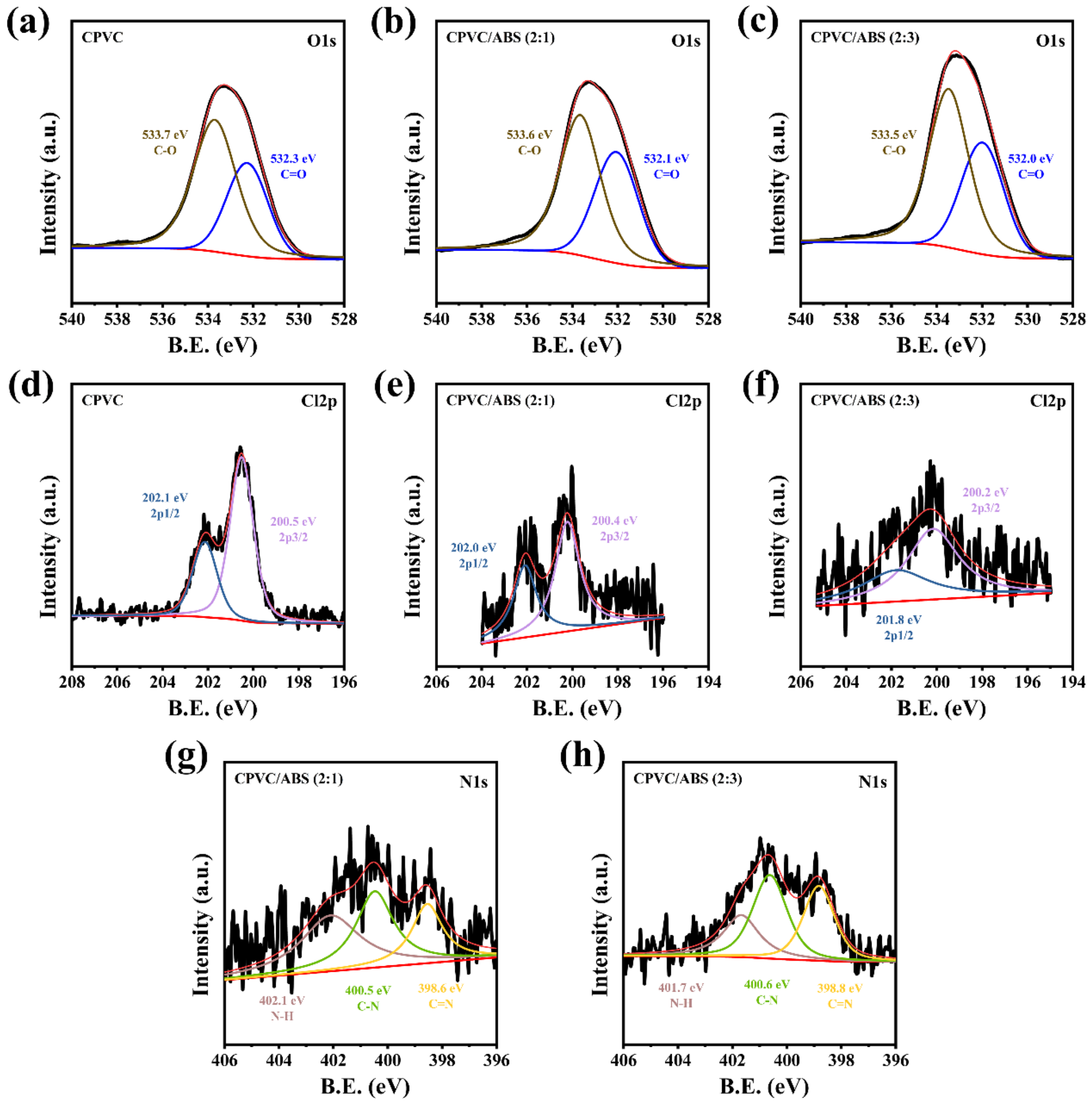

Figure S3. O1s, Cl2p, and N1s regions XPS spectra of residual char of CPVC (a, d), CPVC/ABS (2:1) (b, e, g), and CPVC/ABS (2:3) (c, f, h).

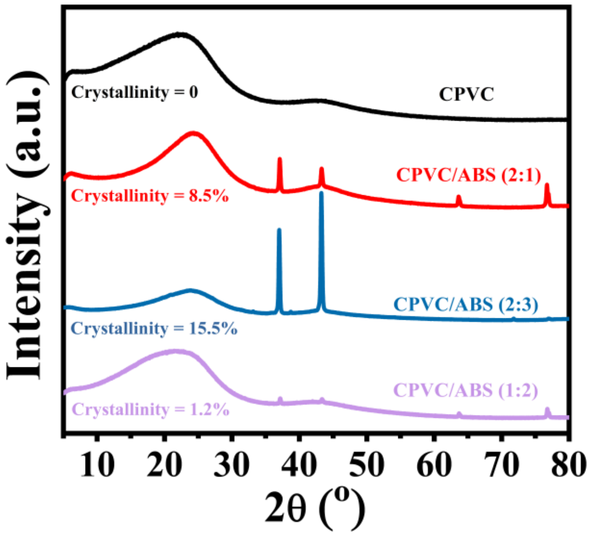

Figure S4. XRD spectra of the char of CPVC, CPVC/ABS (2:1), CPVC/ABS (2:3), and ABS (1:2) composites.

References

1. Goller, S.M.; Schartel, B.; Krüger, S. Phosphorus features halogen –calcium hypophosphite replaces antimony trioxide, reduces smoke, and improves flame retardancy. *Thermochim. Acta* **2024,** *737*, 179764.
2. Yuan, Z.; Wen, H.; Liu, Y.; Wang, Q. Synergy between piperazine pyrophosphate and aluminum diethylphosphinate in flame retarded acrylonitrile-butadiene-styrene copolymer. *Polym. Degrad. Stab.* **2021,** *190*, 109639.
3. Lin, Y.; Dong, W.; Li, S.; Zhang, S.; Chen, X.; Wang, B.; Du, L. Constructing surface oxygen vacancies defect-La-MOF@MXene for enhanced fire safety and smoke suppression properties in robust ABS nanocomposites. *Polym. Degrad. Stab.* **2025,** *234*, 111251.
4. Zhou, F.; Tang, W.; Xi, W.; Qian, L.; Wang, J.; Qiu, Y.; Chen, Y. Improving the fracture toughness, flame retardancy and smoke suppression of ABS by core-shell elastic flame retardant particles with P/Si synergistic effect. *Polym. Degrad. Stab.* **2024,** *228*, 110893.
5. Yildiz, C.; Seki, Y.; Kizilkan, E.; Sarikanat, M.; Altay, L. Development of Halogen-Free Flame Retardant Acrylonitrile Butadiene Styrene (ABS) Based Composite Materials. *Chem. Select* **2023,** *8* (41), e202300989.
